# Retraction Note to: Long non-coding RNA SATB2-AS1 inhibits microRNA-155-3p to suppress breast cancer cell growth by promoting breast cancer metastasis suppressor 1-like

**DOI:** 10.1186/s12935-021-01798-y

**Published:** 2021-02-14

**Authors:** Shaoqiang Cheng, Bingshu Xia, Hongbin Li, Yuying Li, Xinxin Lv, Yue Zhang, Yuanxi Huang

**Affiliations:** 1 Department of Breast Surgery, Harbin Medical University Cancer Hospital, 150 Haping Road, Harbin, China; 2 Department of Medical Oncology, Harbin Medical University Cancer Hospital, 150 Haping Road, Harbin, China

## Retraction to: Cancer Cell Int (2020) 20:321 10.1186/s12935-020-01411-8

The Editors-in-Chief have retracted this article [1]. Concerns were raised with a number of figures, specifically:

- Fig. 3d blank cell culture for MDA-MB-231 and MCF-7 appear identical to Fig. 4d blank cell culture for MDA-MB-231 and MCF-7 respectively.
- Fig. 3d charts appear to be identical to all Fig. 4d charts.
- Fig. 3f blank cell cultures appear to be the same as Fig. 4f blank cell cultures.
- Fig. 3g blank cell cultures appear to be the same as Fig. 4g blank cell cultures.
- Fig. 3i blank tissue samples appear to be the same as Fig. 4i blank tissue samples.

Additionally, the Editors-in-Chief found that blank control values are missing in the raw data for Fig. 4a and that the variation between miRNA mimic and miRNA inhibitor appears to be unrealistically small. The Editors-in-Chief have therefore concluded that the data reported in this article are unreliable.

The authors agree with the retraction but disagree with the wording of the retraction notice.

## References

[1] Cheng S, Xia B, Li H, Li Y, Lv X, Zhang Y, Huang Y (2020). Long non-coding RNA SATB2-AS1 inhibits microRNA-155-3p to suppress breast cancer cell growth by promoting breast cancer metastasis suppressor 1-like. Cancer Cell Int.

